# DataUp: A tool to help researchers describe and share tabular data

**DOI:** 10.12688/f1000research.3-6.v2

**Published:** 2014-09-12

**Authors:** Carly Strasser, John Kunze, Stephen Abrams, Patricia Cruse

**Affiliations:** 1 California Digital Library, University of California Office of the President, Oakland, CA 94612, USA

## Abstract

Scientific datasets have immeasurable value, but they lose their value over time without proper documentation, long-term storage, and easy discovery and access. Across disciplines as diverse as astronomy, demography, archeology, and ecology, large numbers of small heterogeneous datasets (i.e., the long tail of data) are especially at risk unless they are properly documented, saved, and shared. One unifying factor for many of these at-risk datasets is that they reside in spreadsheets.

In response to this need, the California Digital Library (CDL) partnered with Microsoft Research Connections and the Gordon and Betty Moore Foundation to create the DataUp data management tool for Microsoft Excel. Many researchers creating these small, heterogeneous datasets use Excel at some point in their data collection and analysis workflow, so we were interested in developing a data management tool that fits easily into those work flows and minimizes the learning curve for researchers.

The DataUp project began in August 2011. We first formally assessed the needs of researchers by conducting surveys and interviews of our target research groups: earth, environmental, and ecological scientists. We found that, on average, researchers had very poor data management practices, were not aware of data centers or metadata standards, and did not understand the benefits of data management or sharing. Based on our survey results, we composed a list of desirable components and requirements and solicited feedback from the community to prioritize potential features of the DataUp tool. These requirements were then relayed to the software developers, and DataUp was successfully launched in October 2012.

## Note

Much has transpired since we submitted this paper to *F1000Research* in early January of this year. We received funding from the NSF supplemental to the DataONE project that allowed us to hire a developer to continue working on DataUp. Since our completion of DataUp Version 1, Microsoft Research had continued working on the web application version of the tool and had made great strides towards improving its features. Because of this, we partnered with Microsoft Research for the release of DataUp Version 2, which was announced in February 2014 at the International Digital Curation Conference in San Francisco. This release coincided with the retirement of the DataUp add-in for Excel, which is no longer supported. As of July 2014, we are transitioning away from DataUp as an archiving solution for researchers. We will be merging the DataUp tool with our new data sharing platform at the UC3, called Dash.

Dash is a UC-wide project to create a platform that allows researchers to easily describe, deposit and share their research data publicly. Because of the large overlap in functionality between Dash and DataUp, we can provide better support for our users by merging the two projects. The new service will be an instance of our Dash platform, connected to the DataONE repository ONE *Share*. Users will be able to describe their datasets, get an identifier and citation for them, and share them publicly using the Dash tool. The initial implementation of dash.dataone.org will not have all of DataUp’s capabilities for parsing spreadsheets and reporting on best practices compliance. Also while a Dash user can provide dataset-level description using elements of the DataCite schema, column-level (i.e., attribute) metadata will not supporting. However, our intention is to add these missing functions over time as the necessary resources are available.

## Introduction

The move towards digital data is ubiquitous across all domains in academic research and scholarship ^1– 5^, and these data can be made available more easily and distributed more quickly than ever before. This is often called the data deluge, and is a phenomenon that has been examined in the traditional academic literature ^2, 4, 6^, as well as in several major media outlets ^7– 9^.

Among the most pressing problems associated with the data deluge is good data management: how does one handle the huge volume of available information effectively and efficiently to solve important problems? Knowledge of good data management techniques and software development lags behind the progression of the data deluge. Consequently, although researchers of all fields are faced with huge volumes of data from increasingly diverse sources, they do not have the skills to handle their data sets. This challenge is amplified by the fact that research data are seldom shared, re-used, or preserved ^10– 12^. There is a growing awareness among practitioners and funders that this situation represents inefficient use of research dollars, missed opportunities to exploit prior investment, and a general loss for the scholarly community ^13^. Michener *et al.* ^14^ described the loss of valuable data and insight about those datasets as “information entropy”. This loss of information is becoming increasingly worrisome as data management practices improve very slowly, while the volume of data grows exponentially.

Recognizing that most earth, environmental, and ecological scientists use spreadsheets at some point in their data life cycle, the California Digital Library (CDL) partnered with Microsoft Research Connections and the Gordon and Betty Moore Foundation to create a tool that would encourage and enable good data stewardship practices for datasets created in Microsoft Excel. Our vision was to promote publishing, archiving, and sharing of tabular data among earth, environmental, oceanographic, and ecological scientists by creating a tool that will easily integrate into their current workflows and assist them in data management and preservation. This will, in turn, enable faster and more efficient research, thereby increasing the pace of scientific advancement.

Others have worked towards creating tools to help researchers conform to best practices and archive their data. The OpenRefine (formerly Google Refine) project is one such example ( http://openrefine.org). This tool seeks to help researchers work with “messy” tabular data, and is free and open to anyone. However it does not link directly to repositories, and therefore only addressed some of the features we planned to undertake with DataUp. Another related tool for working with spreadsheets is RightField, an open-source tool for adding ontology term selection to Excel spreadsheets ( http://www.rightfield.org.uk). RightField allows researchers to access controlled vocabularies, which results in better quality metadata. Similar to OpenRefine, however, RightField does not have capabilities for archiving research data. To optimize the tool, we first identified the needs of the community via surveys of researchers. We found that, on average, researchers had poor data management practices, were not aware of data centers or metadata standards, and did not understand the benefits of data management or sharing. We used the survey results to compose a list of desirable components and solicited feedback from the community to prioritize potential features.

The resulting DataUp tool facilitates documenting, managing, archiving, and sharing tabular scientific data. It comes in two forms, both open-source: an add-in for Excel and a web-based application. The add-in operates within the well-known program Microsoft Excel; the web application allows users to upload tabular data to the web-based tool in either Excel (.xlsx) or comma-separated value (.csv) format. Both the add-in and the web application provide users with the ability to (1) Perform a “best practices check” to ensure the data are CSV-compatible; (2) Create standardized metadata, or a description of the data, using a wizard-style template; (3) Retrieve a unique identifier for their dataset from their chosen data repository, and (4) Post their datasets and associated metadata to the repository.

## Methods and results

The extent to which researchers use Microsoft Excel is not fully documented, however based on strong anecdotal evidence we assumed that it is a standard tool for most scientists. Given this fact, we determined that an add-in for Excel would have the greatest potential impact on how scientists work with data. An add-in (also called a plug-in) is a small piece of software that one installs on a local computer. Once installed, it extends the capabilities of an existing program: in this case, Excel. The add-in’s functionality is available from within the program, and in the case of Excel, appears as a “ribbon” of functions and features within the standard user interface. In this way, we assumed that researchers would be more likely to use the tool since it is fully integrated with a program they are already using.

Our target audience for creating the tool was scientists and researchers actively working with earth, environmental, oceanographic, and ecological data. These researcher groups were chosen based on their relatively low participation in data sharing ^15^ and their presumed high levels of Excel use. To capture their data management needs, we surveyed and interviewed more than 130 researchers over the course of five months (August to December, 2011). We also collected suggestions for requirements from academic libraries, data centers, data managers, and other data professionals, although this collection was less structured and more anecdotal. Most of these interactions occurred via interactions with the DataONE project community ^16^; a full list of partners affiliated with the DataONE project is available on their website ( http://dataone.org).

### Researcher surveys and interviews

We used several methods to communicate with our potential stakeholder community in developing the tool. These included the DCXL blog (now the Data Pub Blog, located at datapub.cdlib.org), two Twitter accounts (@dataupcdl and @carlystrasser), and interviews and conversations at conferences, webinars, and professional meetings.

Our goal in surveying and interviewing researchers was to determine how they were currently handling data management, especially as it related to Excel data, and how best the tool we were developing might help improve researcher practices surrounding data. The questions we asked underwent revision to improve the survey instrument, and to that end we used four similar versions of the survey over the course of data collection. The number of respondents for each survey version was 43, 12, 47, and 10 respectively, for a total of 112 respondents. The four versions of the survey can be viewed in the associated datasets. Interview questions were less structured and varied depending on the interviewee.

We attended four professional meetings and surveyed researchers of various statuses (i.e., from student to senior researcher) and from many different institutions and organizations ( Table 1). We also conducted surveys and in-depth interviews with researchers at four campuses in the University of California system from September 2011 to February 2012. Interviewees volunteered to participate by contacting one of the authors, Carly Strasser, directly. Overall, we collected 112 surveys and conducted 30 interviews (of 30 to 90 minute duration) from 133 people representing 84 different institutions ( Table 1). Less formal information was obtained from other venues, including comments on the DataUp Blog, discussions with librarians and data center managers, and conversations with researchers at DataONE meetings.

**Table 1.** Locations and events where survey and/or interview data were collected on requirements.

Venue	Collected
Ecological Society of America 2011 Summer Meeting in Austin, TX	55 surveys
American Fisheries Society 2011 Fall Meeting in Seattle, WA	36 surveys
American Geophysical Union 2011 Meeting in San Francisco, CA	10 surveys
Estuarine Research Association 2011 Meeting in Oakland, CA	2 surveys
UCSB	8 surveys, 8 interviews
UC Berkeley	1 survey, 2 interviews
UC Davis	8 interviews
UC Santa Cruz	11 interviews

### Survey results

**Figure 1.**
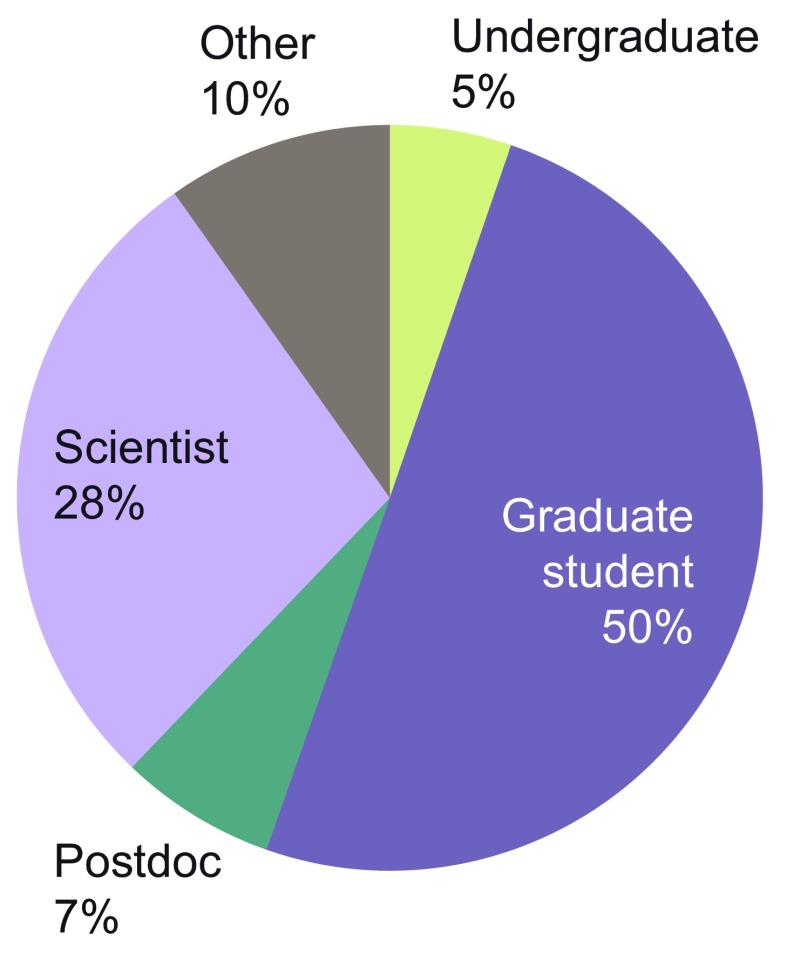
Demographic breakdown of researchers surveyed. *n* = 133.

Demographically, the survey pool was composed of researchers and scientists ranging from undergraduate-level to PhD-level ( Figure 1).

**Figure 2.**
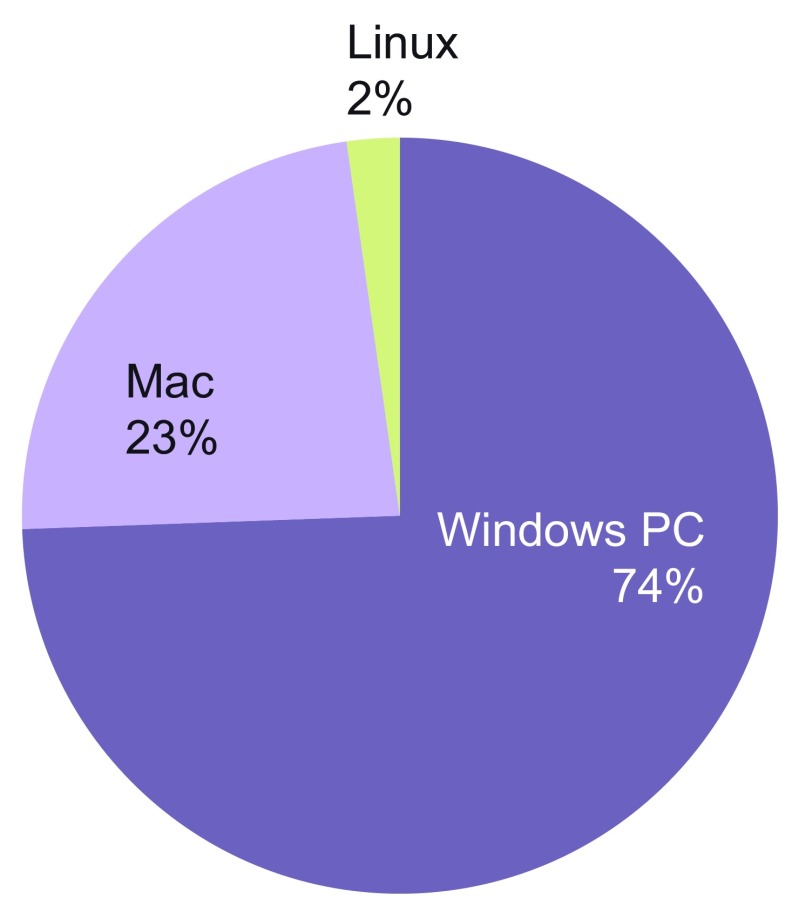
Breakdown of operating systems used by researchers surveyed. *n* = 133.

We asked researchers about their choice of operating system because of the potential implications for development of the tool. Of those surveyed, the large majority (74%) used a Windows-based operating system, while 23% used a Mac-based system and 2% used Linux ( Figure 2).

**Figure 3.**
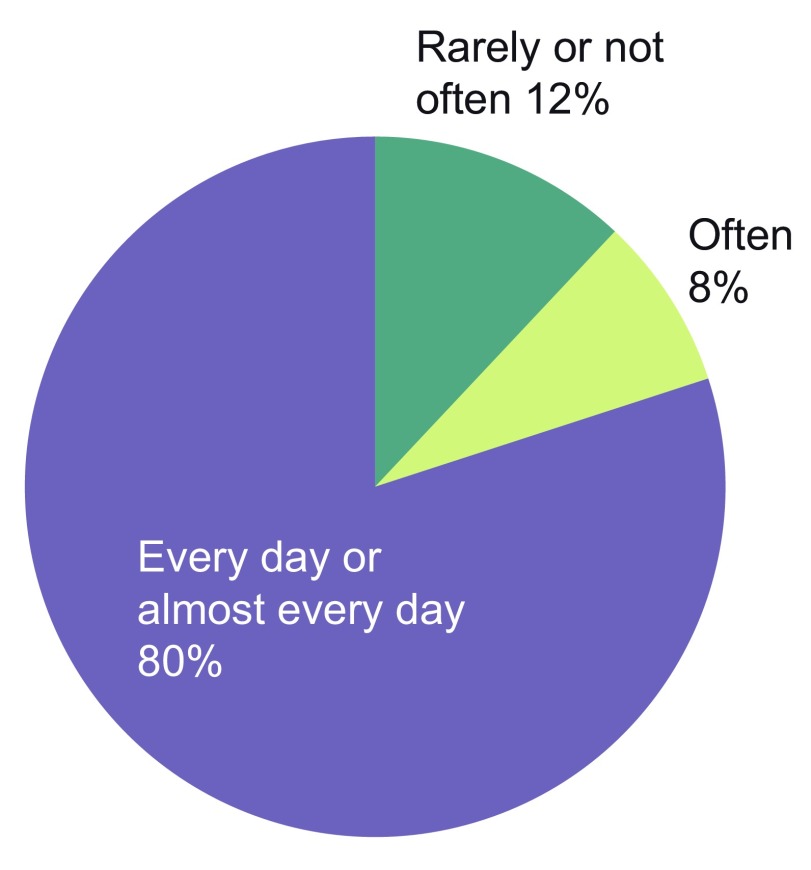
Frequency of Excel use reported by researchers surveyed. *n* = 118.

We asked a series of questions related to how the researchers were using Excel for their day-to-day work. We found that 80% of those surveyed answered that they used Excel “every day” or “almost every day” ( Figure 3).

**Figure 4.**
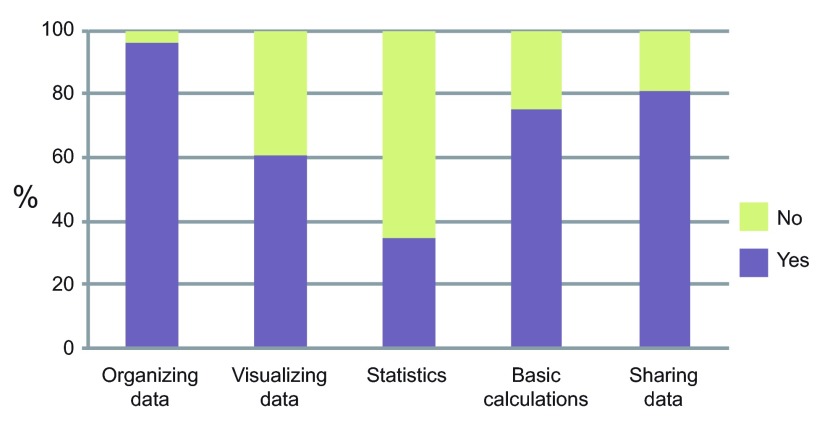
Percent of researchers surveyed who used Excel to perform certain tasks. *n* = 119.

When asked what data-related tasks they were undertaking when using Excel, we found that most were at least using Excel to organize their data (96%). Excel was also used by the majority of participants for visualizing data (61%), performing minor calculations (75%), and for sharing data with colleagues in Excel format (81%) ( Figure 4).

**Figure 5.**
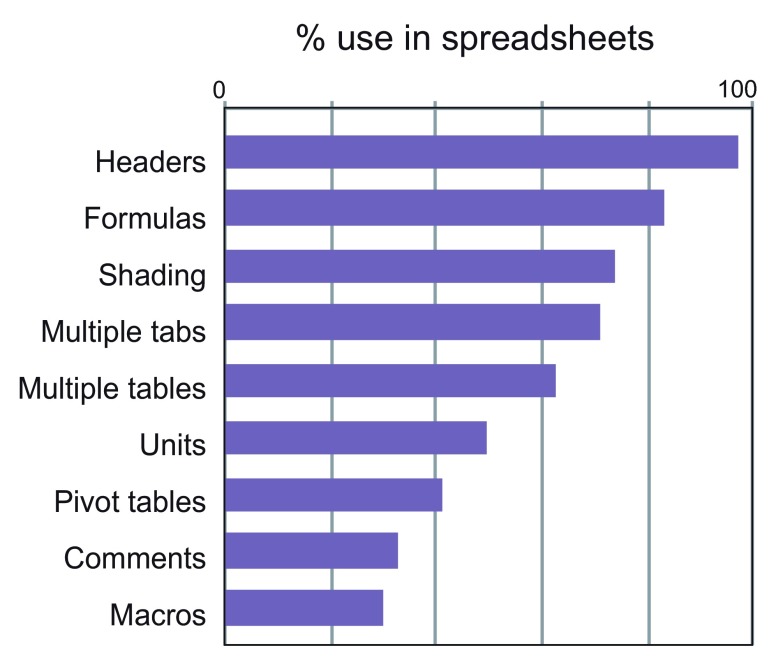
Percent of researchers surveyed that reported a given feature as present in their Excel data. *n* = 70.

To better understand the content of researchers’ spreadsheets, we asked whether the following Excel features were used in their datasets ( Figure 5).

- multiple tables on a single spreadsheet
- multiple tabs within an Excel file
- header row with parameter labels created
- units provided alongside data (i.e., in the data cell or header row)
- embedded formulas
- pivot tables
- macros
- embedded comments
- cell shading to indicate information about the data (i.e., ad-hoc metadata)

Most researchers created header rows (97%), used embedded formulas (83%), and used cell shading as a form of ad-hoc metadata (74%). Of those we surveyed, the majority (74%) reported that they had a “better than average” knowledge of the Excel software, while 24% reported an average knowledge ( *n* = 105).

**Figure 6.**
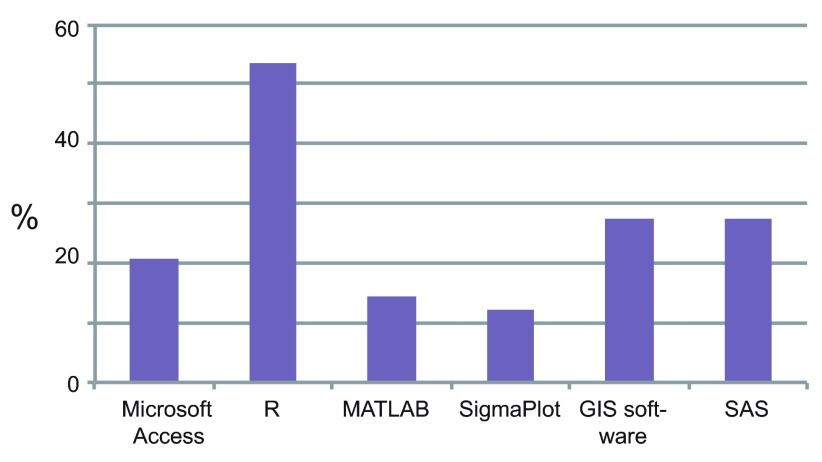
Percent of researchers surveyed that reported using a given program alongside Excel. *n* = 131.

We asked researchers to identify other software programs that they use alongside Excel for their data analysis and organization. Note that these results are likely heavily influenced by the venues used to interview researchers, since software programs tend to be used by many researchers in a given discipline ( Figure 6). The open-source statistical software R was most often cited (53%).

Other information gathered via the survey included areas of work (i.e., field versus lab; area of focus; discipline), attitudes about data sharing, and knowledge of data repositories. These questions were not asked formally via survey in most cases, rendering the results difficult to share with any confidence in the numbers reported.

### Requirements

Although the practices reported by researchers are common and accepted uses of Excel, they are not necessarily well suited for long-term preservation of high-quality data. This has been previously reported in the literature ^17– 20^. In addition, the European Spreadsheet Risk Interest Group has created a curated list of stories detailing instances where spreadsheets are implicated in erroneous reporting ( http://eusprig.org). In general, issues associated with using Excel for generating curation-ready datasets are (1) poor data table construction (e.g. multiple data tables on a single spreadsheet); (2) a lack of metadata or poorly standardized metadata (e.g. using comments, notes, color-coding, and shading to document important details about the dataset; (3) embedded figures, charts, and comments that make the spreadsheet less usable in programs outside Excel; and (4) poor provenance of how data is produced via calculations, statistics, and formulas.

Based on the information collected from researchers and other stakeholders, we created the following high-level requirements for the tool:

1. Check data file for .csv compatibility and create .csv version data file. The user can generate and download a customized report detailing elements in their dataset that might cause problems for data archiving and/or export of the data file as a .csv version.

2. Generate metadata that is linked to the data file. Using the DataUp tool, machine- and human-readable metadata is generated, embedded in the data file, and can be exported as a separate file. The metadata is displayed in a new tab on the spreadsheet, can be saved separately, and relies on Ecological Metadata Language (EML) and the DataONE metadata schema ( http://mule1.dataone.org/ArchitectureDocs-current/design/SearchMetadata.html). Both file-level and parameter-level metadata are created by the tool.

- File-level metadata is information about the entire dataset, such as the creator, temporal and spatial details of the data collection, and the funders of the project. The tool is able to pre-populate some fields based on user information provided by Excel. Keywords can be selected from standard lists.
- Parameter metadata describes individual elements of the data file, and most commonly corresponds to the header row of a tabular dataset. The user can identify a header row to begin the process of creating parameter metadata.

3. Generate a citation for the data file. Using the tool, the user can generate a complete data citation for their tabular dataset. This includes all the metadata necessary for citing the dataset, is in a standard format, and becomes part of the metadata. The citation can be downloaded in standard formats (e.g. .ris, .bib, .xml).

4. Repository authentication set-up. The user can authenticate with their chosen repository from within the tool, assuming they have pre-existing login information for that repository. This will then allow them to deposit their dataset in the repository via the tool.

5. Link an identifier to the data file. The tool allows the user to retrieve and save a persistent identifier (such as a DOI) for their dataset from their chosen repository.

6. Ensure that the data file is ready for deposition into a repository. The tool determines whether the data file is ready for deposit into the designated archive by checking for the following:

- Determine whether a compatibility check has been completed.
- Determine whether metadata is complete (i.e., all required metadata are present).
- Determine whether a citation has been generated.

The tool then generates the technical metadata needed by the designated repository.

7. Submit the data file for deposition into the designated repository.

8. Ensure compatibility for Excel users without the add-in: users without the add-in locally installed are able to open the data file and access the metadata.

These requirements were posted on the DataUp blog, with requests for feedback from the community. We then passed on the document to the Microsoft Research team, who generated a second version of the requirements based on their knowledge of Excel and their protocols for software development. These requirements were then relayed to the developers (contractors for Microsoft Research).

### Add-in versus web-based application

In the course of development, questions arose from the project team as to whether an Excel add-in was the most appropriate choice for delivering the tool to researchers; the alternative discussed was a web-based application. Concerns were that an add-in had compatibility issues that required updates on the developer’s part and downloads on the user’s part. In addition, the project timeline dictated that the add-in could be built only for Windows platforms; Macintosh systems would not be able to use the tool. This is not true for a web-based application. See Table 2 for a summary of the differences between the two potential versions of the tool: an add-in and a web application.

**Table 2.** Feature comparison for the two versions of the DataUp tool: add-in for Excel and Web-based application.

Feature	Excel add-in	Web-based application
Platform compatibility	Windows only	Any
Spreadsheet compatibility	Different add-in for each Excel version	One application covers multiple versions; potential future expansion to SQL, CSV, XML, Open Office, Google Docs etc
Download necessary?	Yes	No
Software updates	Fixed bugs require download & re-install	No download/re-install necessary
Cloud-based?	No	Yes
Offline use?	Yes	No; potential future for HTML5 and offline use
Languages	C#.NET C/C++	HTML/JavaScript C#/ASP.NET
Has all the functionality of Excel	Yes	No

In early 2012, we launched a campaign to determine which of the two versions of the tool should be created. Input was received from attendees of the Ocean Sciences 2012 Meeting in Salt Lake City, Utah. We also asked researchers and others via online surveys and blog posts which they would prefer, and what barriers they perceived to each version of the tool. We collected results from approximately 200 individuals. Most (95%) were willing to download an add-in, and most (83%) indicated that they would prefer an add-in to a web application (assuming the add-in were available for Mac as well). However 72% reported that there were barriers to their downloading and/or installing an add-in for Excel. Barriers mentioned included version compatibility issues, security concerns (e.g., viruses), lack of Mac compatibility, and a lack of administrative controls over computers, preventing downloads. The full set of survey responses is available in the associated datasets.

DataUp manuscript data Data files for F1000Research manuscript submission “DataUp: A tool to help researchers describe and share tabular data”. Authors: C Strasser, J Kunze, S Abrams, P Cruse. Submitted December 2013.
readme.txt has description of all files in this fileset.

Given these contradictory results we determined that there was a need for both versions of the tool. We therefore proceeded with the development of both an add-in for Excel and a web-based application. The requirements were the same for both versions; only the delivery of the functionality differed between the two. Of those surveyed, 75% used a Windows operating system, compared to 22% using a Mac, and 3% using some other system (e.g. Linux). These results paralleled those from our general researcher survey ( Figure 2).

### The DataUp tool

The tool created based on our requirements and user feedback is called DataUp. DataUp is free and open source, and has two forms: a web-based application (web app http://dataup.org) and a downloadable Excel add-in. Both versions of the tool provide users with the ability to (1) perform a “best practices check” to ensure that data are well formatted and organized; (2) create standardized metadata (i.e., a scientifically-meaningful description of the data), using a wizard-style template; (3) retrieve a unique identifier for their dataset from their chosen data repository; and (4) upload datasets and associated metadata to a public data repository.

**Best practices check.** The tool determines whether the data file has any of 11 potential issues that do not comply with data management best practices, such as embedded charts, comments, and color-coded cells. These issues were chosen based on interviews with researchers, as well as data managers who often “clean up” spreadsheets submitted by researchers for archiving. In addition to identifying the locations of these problems, DataUp informs the user why they are potentially problematic, and offers suggested alternatives or the ability to remove them in bulk. The information provided by the DataUp tool for each of these potential issues is below:

1. Embedded charts, tables, pictures. **Why:** These embedded items will not be visible when data are exported as a .csv file. Also, these elements are visible only if the file is opened with Microsoft Excel. **Suggested remedy:** Move embedded charts, tables, or pictures to other tabs in your file or to a completely separate file.

2. Embedded comments. **Why:** Comments will not be visible when data are exported as a .csv file. Also, these elements are visible only if the file is opened with Microsoft Excel. **Suggested remedy:** Create a new column titled “Comments” and add your text there.

3. Commas. **Why:** Commas are often used to separate multiple piece of information/data (e.g. City, State). Cells only contain one piece of information. **Suggested remedy:** Split pieces of information into multiple columns (e.g. City column and State column).

4. Special characters. **Why:** Special characters may cause problems for other programs or may be modified upon export. **Suggested remedy:** Use alpha-numeric characters only. If needed, describe the symbol in a new column.

5. Color coded text or cell shading. **Why:** Formatting will not be visible when data are exported as a .csv file. If formatting is used as a coding scheme, all codes will be lost upon export. **Suggested remedy:** Use descriptions or alphanumeric coding schemes in a new column.

6. Columns have mixed data types. **Why:** Some programs cannot handle mixed data types (e.g. numbers and text in the same column). **Suggested remedy:** Ensure you are using only numbers or only text in a column; split data into multiple columns if necessary.

7. Non-contiguous data. **Why:** Empty columns or rows tend to be used to separate multiple data tables on the same tab. **Suggested remedy:** Move multiple tables onto separate tabs.

8. Merged cells. **Why:** Merged cells will not be maintained when data are exported as a .csv file. Information may be lost when cells are un-merged upon export. **Suggested remedy:** Un-merge cells and annotate appropriately so information is not lost.

9. Blank cells. **Why:** Blank cells within a contiguous data table are potentially problematic for reading files in other programs. **Suggested remedy:** Designate a coding scheme for missing data or other explanations for blank cells.

10. Header row absent or more than one header row. **Why:** Ideally the first row of a data table contains parameter names for the columns. If there is no header row, your data table may be difficult to use and document. If there are multiple header rows, some software programs may have problems. **Suggested remedy:** Create a header row with unique parameter names that describe the column’s contents.

11. Multiple sheets (tabs). **Why:** Multiple sheets will not be maintained as a single document if the file is converted to .csv. **Suggested remedy:** The user can move each tab into a separate .csv file. If left as multiple sheets, the DataUp tool will automatically export the data as separate .csv files.

**Create metadata.** DataUp helps the researcher create standard metadata using a form that becomes part of their spreadsheet, facilitating future use and sharing. Metadata can be generated at both the file- and column-level. File-level metadata includes names, email addresses and institutional affiliations for project personnel, and dataset titles. Column-level metadata (i.e. attribute metadata) includes information about the variables in the dataset, the units of measure, and descriptions of each column of data. DataUp creates metadata using the Ecological Metadata Language (EML). This particular standard was chosen because of its widespread use in our original target communities. In addition, EML is both flexible and extensible, which enables future modifications to the chosen schema as necessary. We selected 47 elements of EML for DataUp, with seven elements required ( Table 3). We choose to support only a subset of EML in order to provide the lowest barrier to entry for researchers interested in documenting their datasets.

**Table 3.** Metadata elements chosen for the DataUp metadata schema. *elements are required.

*Basic Information* Today’s date* Title of dataset* Keyword thesaurus used Formatted citation	Abstract* Keyword(s)* Identifier
*Information about Personnel* Creator: First name* Creator: Organization Creator: City Creator: Postal code Creator: Phone Data Contact Person: First name Data Contact Person: Organization Data Contact Person: City Data Contact Person: Postal code Data Contact Person: Phone	Creator: Last name* Creator: Address Creator: State/province Creator: Country Creator: Email* Data Contact Person: Last name Data Contact Person: Address Data Contact Person: State/province Data Contact Person: Country Data Contact Person: Email
*Information about the Dataset* Temporal coverage: Beginning date Geographic coverage: Description East bounding coordinate South bounding coordinate Project title Project personnel Data Publisher: repository name Data table description	Temporal coverage: Ending date West bounding coordinate North bounding coordinate Intellectual rights Project description Project personnel role Data table name

**Obtain an identifier.** Valuing and incentivizing the time and effort required to manage data well is an important factor in fostering data sharing and reuse. One way to allow data producers to get credit for this is through data citation. The DataUp tool connects to the user’s chosen repository to retrieve a unique identifier for the researcher’s dataset. For its first iteration, DataUp connects to the EZID service ( http://n2t.net/ezid), based at CDL, used by the public DataUp ONE *Share* repository. The identifier generated is an ARK (Archival Resource Key, https://confluence.ucop.edu/display/Curation/ARK). ARKs provide stable, opaque, versatile, and transcription-safe identifiers. This identifier is saved in the data file’s metadata.

**Share and archive.** Once metadata is created, the user can connect directly to a repository via DataUp and upload their data for archiving. Currently, DataUp is connected to ONE *Share*, which is a dedicated public DataUp repository to which anyone may deposit tabular data (more information below).

## Architecture

**Figure 7.**
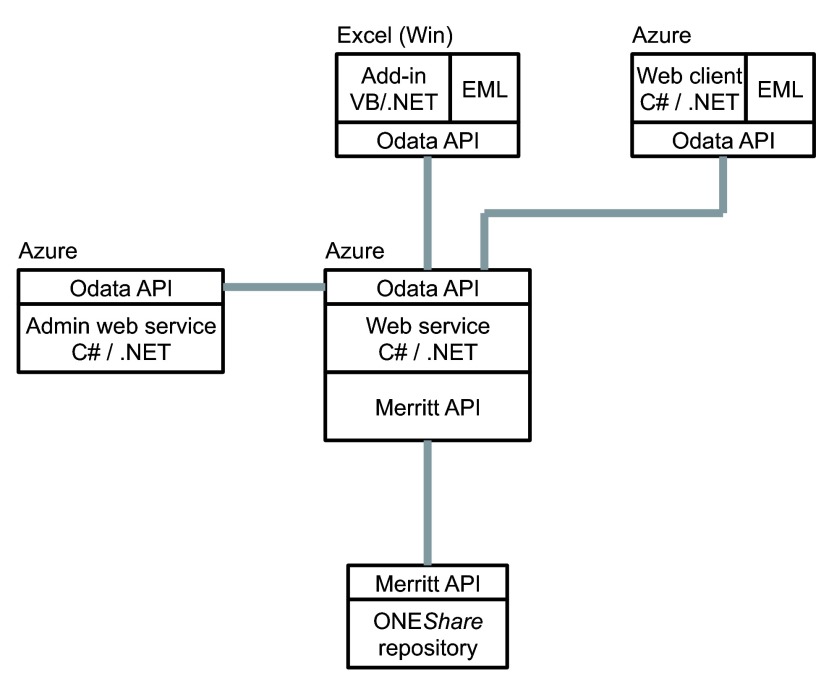
Architecture of the DataUp web service, web application, and add-in for Excel, and how they relate to the ONE *Share* repository.

DataUp’s codebase is written in C# using the .NET application framework. The web app is deployed on Microsoft’s Windows Azure cloud platform. DataUp’s architecture ( Figure 7) consists of two clients communicating via an intermediating web service to one or more repositories. The add-in client is an Excel extension that runs directly on a researcher’s Windows-based computer. The web app client runs as an online application hosted in Azure. Client/web service communication uses the OData protocol ^21^. Both clients support standard EML metadata and draw functionality from a common web service, also hosted in Azure. That web service is managed by a separate administrative service.

DataUp was designed not only for standalone metadata checks, but also for contacting a variety of repositories to obtain persistent identifiers and to archive data. Currently, the only repository supported is ONE *Share*, an instance of the CDL Merritt repository that is also a DataONE Member Node (more information below). With the front-end running at CDL and a storage node back-end running at the University of New Mexico, content can be browsed either by logging in directly to Merritt as a guest or using the DataONE ONE *Mercury* interface ( http://dataone.org/onemercury).

### Creation of the ONE *Share* repository

Although there are hundreds of data repositories available to researchers for data archiving, the majority of scientists are not aware of their existence or how to access them. One of the major outcomes of the DataUp project is the ONE *Share* repository, created specifically for the DataUp tool. ONE *Share* is a special instance of CDL’s Merritt repository, which serves as a digital archive and access system to the University of California campuses ( http://www.cdlib.org/uc3/merritt). Users can deposit their tabular data and metadata directly into the ONE *Share* repository from within the tool, allowing for seamless data archiving within the researcher’s current workflow. The DataUp web service performs the repository submission using the Merritt API, hiding all details of the transfer protocol from the DataUp user. An added advantage of ONE *Share* is its connection to the DataONE network of repositories. DataONE links together existing data centers and enables its users to search for data across all participating repositories using a single search interface. Since Merritt is a member node on the DataONE network, all data deposited into ONE *Share* will be indexed and made discoverable by any DataONE user, facilitating collaboration and enabling data re-use.

The ONE *Share* repository is collaboratively supported by the CDL and the University of New Mexico Library. CDL’s Merritt repository relies on a highly decentralized micro-services architecture ^22^. In the case of ONE *Share*, a Merritt storage node was established on a University of New Mexico (UNM) virtual server managing a local file system. All DataUp submissions to Merritt are routed automatically to the UNM storage node, but the data are still subject to all Merritt preservation and access services such as ongoing fixity audits, metadata search and browse, and pro-active preservation analysis and planning. Merritt is also integrated as a member node on the DataONE network and the full set of descriptive metadata for all DataUp-submitted data is automatically harvested by the DataONE coordinating nodes for inclusion in the federated ONE *Mercury* search interface, increasing the public visibility of DataUp datasets.

### Beta testing/feedback

The first versions of the add-in and web application underwent beta testing by researchers, librarians, software engineers, and other stakeholders. Testers included professional contacts of the DataUp team, researchers who participated in the requirements-gathering survey and consented to future contact, and individuals responding to a blog post requesting subjects for beta testing. We received feedback from 23 testers via an online survey. We received additional comments via email and conversations with researchers. Information gathered from the beta testers was relayed to the developers who addressed those issues that were reasonable within the given time frame for software release. Data from beta testing is available from the associated datasets.

### Formation of a community

One of the major goals of the DataUp project was to create an open-source tool that could be adopted and used by the larger community. To that end, we partnered with the non-profit Outercurve Foundation, whose goal is to enable code exchange and understanding among software companies and open source communities. The DataUp project site for Outercurve holds the copyright to DataUp code, and has released it under Apache2.0 license ( https://www.outercurve.org/Galleries/ResearchAccelerators/DataUp). The code for all aspects of the DataUp tool (add-in, web app, and web service) is available on the project’s BitBucket site ( http://www.bitbucket.org/DataUp). Minimum system requirements for the web application are an internet connection and web browser. For the add-in, the user must be running a Windows operating system with Microsoft Excel 2007 or higher.

## Discussion and conclusions

### DataUp success

Response to the release of the tool was enthusiastic. Between October 2012 and December 2013, the add-in version of the tool had been downloaded more than 700 times, and we estimate a proportionate interest in the web app version of the tool. The main DataUp website has had over 17,000 page views with visitors from more than 10 countries (84% of visits from the US). These numbers do not, however, adequately represent the tool’s popularity and potential. The CDL has received inquiries about DataUp from many repositories, organizations, and publishers interested in configuring the tool for their needs. The inquiries represent a range of stakeholders that are crucial to data sharing, including a large citizen science project, a major social science data archive, some high-profile data publication services, and others. They are excited about the possibilities that DataUp represents for linking researchers’ workflows directly to repositories, with capabilities for generating metadata and performing best practices checks.

### Future plans

The DataUp team received a one-year grant from the US National Science Foundation, supplemental to the DataONE project. Using these funds, the DataUp web application will undergo another iteration that will result in easier repository connections, better features, and a more streamlined workflow. The code resulting from this project will be open-source, and community ownership will be encouraged. The text of the NSF proposal is available from the University of California’s eScholarship repository ^23^.

CDL envisions that the future of DataUp will be directed by the community of stakeholders. Interested developers can expand upon and increase the tool’s functionality to meet the needs of a broad array of researchers. Code for both the add-in and web application is open source and participation in its improvement is strongly encouraged. Although the target audience for our tools that result from the DataUp project will be earth, environmental, oceanographic, and ecological scientists, we envisage that any tools developed will be easily implemented in other research communities, such as the social sciences.

## Data and software availability

### Data

Figshare: DataUp manuscript data, doi: 10.6084/m9.figshare.884625 ^24^.

### Software

Zenodo: The DataUp source code package, doi: 10.5281/zenodo.7639 ^25^.

Bitbucket: Source code for the DataUp Excel add-in and web application, https://bitbucket.org/dataup/.
