# Supplementary material for: DataUp: A tool to help researchers describe and share tabular data
Source: F1000Res. 2014 Sep 12;3:6. Originally published 2014 Jan 9. [Version 2] doi: 10.12688/f1000research.3-6.v2 (PMC4304223; doi:10.12688/f1000research.3-6.v2)
Supplement: DataUp manuscript data — Data files for F1000Research manuscript submission “DataUp: A tool to help researchers describe and share tabular data”. Authors: C Strasser, J Kunze, S Abrams, P Cruse. Submitted December 2013. readme.txt has description of all files in this fileset. [file f1000research-3-5502-s0000.tgz › addinVwebapp_Survey.pdf]

DCXL Scientist Survey

1. Please indicate your status:

- ○ Undergraduate
- ○ Masters Graduate student
- ○ PhD Graduate student
- ○ Postdoctoral researcher
- ○ Scientist (Bachelors level)
- ○ Scientist (Masters level)
- ○ Scientist (PhD level)
- ○ Other (please specify)

2. What percent of time do you work on your Excel spreadsheets (or data in general) offline?

% OFF line

% ON line

3. Please answer the following:

	Yes	No
Have you ever used an Excel add-in? This would require that you download the add-in from a website. After downloaded, the capabilities of Excel would be extended.	○	○
Would you be willing to download an Excel add-in that facilitates data management?	○	○

4. Are there any barriers to you downloading and using an Excel add-in? If yes, please describe.

5. What platform do you primarily use with Excel?

- ○ Mac
- ○ Windows
- ○ Other (e.g. Linux)

6. How likely are you to return to a web application to do each of the following tasks? 1 = very likely; 5 = not likely at all

	1	2	3	4	5
Create a metadata tab in your spreadsheet	○	○	○	○	○
Check your metadata for errors	○	○	○	○	○
Check your spreadsheet for compatibility with a data repository	○	○	○	○	○
Connect to a data repository	○	○	○	○	○

7. Which of these scenarios for using DCXL is more desirable?

- ○ Download and install an Excel add-in
- ○ Go to a web-based application and upload your spreadsheet

8. In terms updating software, Which of these scenarios is more desirable?

- ○ Download and install Excel add-in, then download updates as necessary
- ○ Connect to web application that is automatically updated (no download neccesary)

9. Are you more likely to use a web application If there is a hyperlink embedded within Excel?

- ○ Yes
- ○ No

10. Additional thoughts or comments:
