# Supplementary material for: DataUp: A tool to help researchers describe and share tabular data
Source: F1000Res. 2014 Sep 12;3:6. Originally published 2014 Jan 9. [Version 2] doi: 10.12688/f1000research.3-6.v2 (PMC4304223; doi:10.12688/f1000research.3-6.v2)
Supplement: DataUp manuscript data — Data files for F1000Research manuscript submission “DataUp: A tool to help researchers describe and share tabular data”. Authors: C Strasser, J Kunze, S Abrams, P Cruse. Submitted December 2013. readme.txt has description of all files in this fileset. [file f1000research-3-5502-s0000.tgz › Surveys.pdf]

**Status** (circle one)	Undergrad student<br>Other:	Masters grad student	PhD grad student	Postdoc	Masters level scientist	PhD level scientist
**Area?** (Circle all that apply)	Field	Laboratory	Theoretical	Modeling	Experiments	Observations
	Aquatic	Terrestrial	Plants	Animals	Microbes	Cellular
**What system do you use?**		Mac	PC	Linux/Unix		
**Do you use Excel in the course of your research?**		Yes	No			
**If Yes, to what extent?**		Rarely				Every Day
		1	2	3	4	5
**Do you use other spreadsheet programs?**		Open Office	Google spreadsheets	Other:		
**How do you use Excel?** (Circle all that apply)		organize data	visualize data	statistics	other types of analysis	share data
	Other:					
**How knowledgeable are you in Excel?**		Not at all				Very
		1	2	3	4	5
**Do you use Excel with other programs?**		Yes	No			
What programs?						
**Do you share data using Excel?**		Yes	No			
Name						
Email						
Affiliation						
Can I contact you in the future regarding this survey?			Yes	No		
Prefered method of contact:			Email	Phone	Video Conference	Chat

Contact info if not email:		
Would you like to be added to the email list for the DCXL project?	Yes	No

**Status** (circle one)	Undergrad student<br>Other:	Masters grad student	PhD grad student	Postdoc	Masters level scientist	PhD level scientist
**Area?** (Circle all that apply)	Field<br>Aquatic	Laboratory<br>Terrestrial	Theoretical<br>Plants	Modeling<br>Animals	Experiments<br>Microbes	Observations<br>Cellular
**What platform do you use?**		Mac		PC		Linux/Unix
**To what extent do you use Excel?**		Rarely<br>1	2	3	4	Every Day<br>5
**Do you use other spreadsheet programs?**		Open Office		Google spreadsheets	Other:	
**How do you use Excel?** (Circle all that apply)	Other:	organize data	visualize data	statistics	other types of analysis	share data
**What programs do you use along with Excel?**						
**How knowledgeable are you in Excel?**		Not at all<br>1	2	3	4	Very<br>5
**Do you know what metadata are?**			Yes	No		
**Describe your average spreadsheet:**			Tables: 1		>1	
pivot tables			Tabs: 1		>1	
headers		macros		comments		
units		embedded metadata		controlled vocabulary		
formulas		formatting as organization		companion metadata file		
**Do you know of any repositories for your data?**						
**Have you shared data publicly?**						
**How much work would it be to get your spreadsheet into a form useable by others?**		A little<br>1	2	3	4	A lot<br>5
Name						

Affiliation		
Email		
Can I contact you in the future regarding this survey?	Yes	No

Carly Strasser
carly.strasser@ucop.edu

**Status**	Undergrad student	Masters student	PhD student	Postdoc	Masters level scientist	PhD level scientist	
**What does your research involve?**			Field work	Lab work	Models	Experiments	
**When using Excel, what platform do you primarily use?**				Mac	PC	Linux/Unix	
**How Frequently do you use Excel?**	Rarely	1	2	3	4	5	Every Day
**How knowledgeable are you with Excel?**	Not at all	1	2	3	4	5	Very
**What other spreadsheet programs do you use?**	Open Office	Google Spreadsheets	Access	Libra			
	Other:						
**What do you do with Excel? (circle all that apply)**	organize data	visualize data	statistics	simple calculations	share data with colleagues		
**What software do you use along with your Excel spreadsheets for data processing and/or analysis?**	R	SigmaPlot	GIS/ArcGIS	Systat	Canoco		
	Matlab	SAS/JMP	Primer	PC-ORD	Statview		
	Other:						
**Define metadata:**							
**Describe your average spreadsheet:**			Descriptive header rows	pivot tables	comments		
# Tabs per Excel file:	1	>1	Units indicated	macros	cell shading		
# Independent tables per sheet:	1	>1	Embedded formulas	Metadata in separate file	Metadata in same Excel file		
**What would you most like an Excel add-in to help you do?**	manage & organize data better for *my own use*	manage & organize data better for *others to use*	Create archive-specific metadata				
	manage & organize data better for *easier archiving*	Help create project-level metadata (locations, investigators, dates)	help me publish my data publicly				
**What repositories are you aware of to which you could submit your data?**							
**How important is it to share data publicly? (not password protected)**	Not at all	1	2	3	4	5	Very
**How much work would it be to get your spreadsheet(s) into a form useable by others?**	Very little	1	2	3	4	5	Many hours
Name							

Affiliation			
Email	Can I contact you re. this survey?	Yes	No

**Highest degree earned**	High School	Associates	Bachelors	Masters	PhD		
**Affiliation**	Government	Academic	Museum	NGO	Other:		
**What does your research involve?**	Field Work<br>Lab Work<br>Data analysis only	**Excel version?**	Mac<br>PC	2000<br>2000	2001<br>2002	2004<br>2003	2008 / 2011<br>2007 / 2010
**How Frequently do you use Excel?**	Rarely	1	2	3	4	5	Every Day
**How knowledgeable are you with Excel?**	Not at all	1	2	3	4	5	Very
**What other spreadsheet programs do you use?**	Open Office	Google Spreadsheets	Libre				
**What software do you use for data processing and/or analysis?**	R<br>Matlab	SigmaPlot<br>SAS/JMP	GIS/ArcGIS<br>Access	Other:			
**Describe your workflow**							
**Describe your analysis**							
**Metadata definition?**	**Where is your metadata?**	Separate Excel file<br>Same Excel file, different tab<br>Same Excel file, same tab	Lab notebook<br>Other computer file:<br>Other:				
**How do you handle versioning?**							
**How do you back up your data?**							
**What repositories are you aware of to which you could submit your data?**							
**How likely are you to publicly share your data after publication?**	Not at all	1	2	3	4	5	Very
**How much work would it be to get your spreadsheet(s) into a form useable by others?**	Very little	1	2	3	4	5	Many hours
Name	Affiliation						
Email	Can I contact you re. this survey?	Yes	No				
