# Supplementary material for: DataUp: A tool to help researchers describe and share tabular data
Source: F1000Res. 2014 Sep 12;3:6. Originally published 2014 Jan 9. [Version 2] doi: 10.12688/f1000research.3-6.v2 (PMC4304223; doi:10.12688/f1000research.3-6.v2)
Supplement: Peer review history

# Peer review: Referee response for version 2

**DOI:** 10.5256/f1000research.5502.r6119

**Published:** 2015-01-12

**Authors:** Carole Goble

**Affiliations:** 1 School of Computer Science, University of Manchester, Manchester, UK

The revisions and responses by the authors are fair, given their constraints as outlined. The new work with DASH is exciting.

I would still like more thorough related work - ISATools and Ontomaton are in this space and should be discussed. Rightfield does work with an archive system (the SEEK)  n the same way that DataUP works with dataONE and will work in DASH approach. see doi:10.1007/978-3-642-41338-4_14

I have read this submission. I believe that I have an appropriate level of expertise to confirm that it is of an acceptable scientific standard.

# Peer review: Referee response for version 1

**DOI:** 10.5256/f1000research.3427.r5385

**Published:** 2014-07-09

**Authors:** Louise Corti

**Affiliations:** 1 UK Data Archive, University of Essex, Colchester, UK

This paper describes, in some detail, the development, implementation and piloting of a data documentation tool for tabular data.Overall the paper is very clear and provides an easy-to-read narrative of why and how the tool was developed, and how it can be used, as well as tips on populating and handling spreadsheet data, so the data has a longer-life.I am impressed with the simplicity of this tool, which attempts to solve issues in data description for a single type of data. This is much better than the 'workbench' approaches that try to do too much and end up failing.The issue of errors in data conversion between formats is critical and is a known issue in the data archival world. This tool addresses some of these common issues that arise in both spreadsheet data description and conversion.The paper presents some very useful information gathered from surveys and pilot work, but this is rather US-centric.  I don't imagine the use cases of these type of scientists' behaviour in other countries are that different, but I would expect some pointers to this wider context.The referenced literature is good and covers many of the key sources I would refer to. However my own organisation in the UK has been advising on data documentation, including use of Excel and conversion issues, for some years, so it would be good to cite some examples of other efforts to address these issues on the non-ecology field and offer examples of non US resources that provide extensive data management advice ( http://ukdataservice.ac.uk/manage-data.aspx).On page 6 the checklist of issues is very clear and useful and great to alert researchers to these issues upfront.In terms of platforms for the tool, I think a Mac version will be important. In my experience, many data creators prefer to have the convenience of local tools to document data, rather than relying on web-based tools, that can suffer from browser issues and loss of data through poor connection.I do believe that data preparation tools are best built into researchers' existing data handling software, as this brings the activities a step closer to data analysis and away from the burden of completing data deposit forms.I love the idea that the source code has been made available and that, on the whole, the  project has been carried out in the spirit of openness, despite using a Microsoft base for the tool.I am also terribly impressed with the work done to convince Microsoft of the importance of this tool, and to secure codevelopment to enable it to be a plug-in.  On this front, I have had some negative experience in lobbying software suppliers of qualitative analysis packages to implement a data exchange standard to enable export of within-system documentation; conversion between different market leaders' softwares is currently difficult, if not impossible. They should possibly take a leaf out of Microsoft's book and also listen to what data archivists/publishers are saying! The tool looks like it has had some user testing and feedback. Overall I believe this tool could have much wider value than the purposes for which the team have developed it. By simply replacing the metadata standard in use it could easily be applied to other disciplines, e.g. social science data. I would be very keen to pilot it and offer feedback on our own tabular data collection in the social sciences domain. The social sciences use the Data Documentation Initiative (DDI) which has fields that map pretty close to the schema used in the tool and discussed in this paper.  I would advocate engagement with more data centres, possibly through forums like the Research Data Alliance.

I have read this submission. I believe that I have an appropriate level of expertise to confirm that it is of an acceptable scientific standard.

# Response

**Published:** 2014-08-18

**Authors:** Carly Strasser

**Reviewer text is italicized; our responses are below the reviewer text.**

*The paper presents some very useful information gathered from surveys and pilot work, but this is rather US-centric.  I don't imagine the use cases of these type of scientists' behaviour in other countries are that different, but I would expect some pointers to this wider context.*

In retrospect, it would have been valuable to collect use cases and feedback from non-US researchers. We were limited somewhat by our 12 month timeline and focused primarily on the communities that we could easily interview given our spatial and temporal constraints. Based on conversations with our international colleagues, it is our experience that researcher behaviors, concerns, and use cases are, across the board, fairly similar to what we found.

*However my own organisation in the UK has been advising on data documentation, including use of Excel and conversion issues, for some years, so it would be good to cite some examples of other efforts to address these issues on the non-ecology field and offer examples of non US resources that provide extensive data management advice (* *http://ukdataservice.ac.uk/manage-data.aspx* *).*

We recognize that many groups work in the area of best practices for data management. We did not, however, choose to focus on this aspect of community involvement since it has been largely covered by others. That said, we have added a paragraph referencing other projects that have similar goals to DataUp, one of which is based in the UK.

*In terms of platforms for the tool, I think a Mac version will be important. In my experience, many data creators prefer to have the convenience of local tools to document data, rather than relying on web-based tools, that can suffer from browser issues and loss of data through poor connection.*

We concur that a Mac version of the tool would be quite valuable.  However, given our limited budget and abbreviated schedule, it was not possible for the first iteration of DataUp.  Also, the fundamentally different architectures of the Mac and Windows versions of Excel meant that a Mac plug-in would have had to been created from scratch with no opportunity for code re-use from the Windows version.  We hope that interested members of the open source will take on this task in the near future.  We would be happy to support any such effort to the fullest extent possible.

*Overall I believe this tool could have much wider value than the purposes for which the team have developed it. By simply replacing the metadata standard in use it could easily be applied to other disciplines, e.g. social science data. I would be very keen to pilot it and offer feedback on our own tabular data collection in the social sciences domain. The social sciences use the Data Documentation Initiative (DDI) which has fields that map pretty close to the schema used in the tool and discussed in this paper.*

It’s true that the value of the tool goes well beyond just our target audience for requirements development and feedback. Social scientists in particular have expressed interest in the DataUp tool as a potentially valuable addition to their toolkit.  One of the advantages of the DataUp/Dash merger is that the Dash platform has a more open architectural design that will greatly facilitate the process of supporting alternative metadata schemas.

*I would advocate engagement with more data centres, possibly through forums like the Research Data Alliance.*

We also agree that engaging more data centres would be ideal. The RDA was not yet formed when this work was conducted, but moving forward we hope to take advantage of such coalitions to get more uptake of a tool like DataUp.

# Peer review: Referee response for version 1

**DOI:** 10.5256/f1000research.3427.r4573

**Published:** 2014-05-02

**Authors:** Carole Goble, Katy Wolstencroft

**Affiliations:** 1 School of Computer Science, University of Manchester, Manchester, UK; 2 Leiden Insitute of Advanced Computer Science, Leiden University, Leiden, Netherlands

The paper describes the DataUp Excel-based metadata and data capturing tool developed as part of the DataONE project. I like the paper and I like the tool.

- The paper is well written.
- The need for spreadsheet-based data management tools is critical, and DataUp makes a valuable contribution.
- The tool works, the software is available, is being used, and is useful.
- The survey results and the requirements are a very useful guide for other workers using spreadsheets as a prime mechanism for data upload. The survey is well conducted given the constraints of such things, and it is refreshing to see that the people who do the data management (postdocs, postgrads) were targeted.  This is a very useful contribution to the field.

There are, however, some improvements that I would like to see in the final article:

- The metadata model seems to be very high level (Table 3). Is there a richer metadata model for specific data types? Is it possible to upload and index/search on more domain specific metadata captured in the DataUp model? To what extent does the metadata model work for all the data types you mention? As your user base is wide one would expect heterogeneity to be a big problem.
- How are controlled vocabularies and or specific domain metadata models incorporated? The architecture figure 7 is a very general figure and can be replaced by something that showed the protocol of how DataUp is used in practice. Are Excel templates prepared by the DataUp team or through the Plug-in?
- There is sparse information on uptake or the impact of uptake. The number of downloads are listed but not how many datasets were uploaded to the repository using DataUp.
- Despite the excellent requirements survey and user engagement, there is no evaluation of DataUp’s use. What is the difference in uptake between the Excel and web-based version?
- There is no related work section. The only related work is RightField (reference17) but what RightField does and how it relates to DataUp is not mentioned. Similar tools to DataUp such as ISAtool Suite and Ontomaton are not mentioned.
- The survey appears USA-centric. EZID is only available in the USA. DataUp currently works with DataONEShare. Can and how DataUp be adapted for use in other repositories? Can it reuse infrastructure that is not US based? 

**Very minor comments:**

- There are two typos:
  - sheet,s -> sheets
  - highprofile -> high profile

We have read this submission. We believe that we have an appropriate level of expertise to confirm that it is of an acceptable scientific standard, however we have significant reservations, as outlined above.

# Response

**Published:** 2014-08-18

**Authors:** Carly Strasser

**Reviewer text is italicized; our responses are below the reviewer text.**

*The metadata model seems to be very high level (Table 3). Is there a richer metadata model for specific data types? Is it possible to upload and index/search on more domain specific metadata captured in the DataUp model? To what extent does the metadata model work for all the data types you mention? As your user base is wide one would expect heterogeneity to be a big problem.*

Currently there is not a richer metadata model. Future development plans include the ability to expand metadata options and allow for generating metadata files that comply with different standards for various disciplines. We chose some elements of EML as our baseline for three reasons: (1) the metadata fields are fairly generic, so they can apply to many different disciplines; (2) EML is a flexible metadata language that, although originally constructed for Ecology, has the ability to describe a wide variety of datasets; and (3) EML is one of the metadata standards accepted by the DataONE network.

*How are controlled vocabularies and or specific domain metadata models incorporated? The architecture figure 7 is a very general figure and can be replaced by something that showed the protocol of how DataUp is used in practice. Are Excel templates prepared by the DataUp team or through the Plug-in?*

There are no controlled vocabularies incorporated into the tool, although this has been on our “wish list” of future development. Currently a user can specify a controlled vocabulary in the metadata, however we have no connection or integration with vocabulary systems (and no current mechanism for that connection). Similarly, the metadata must be hard-coded and therefore we have no ability to “switch out” the metadata depending on domain-specific interests.

There are no templates in use by the DataUp tool. Instead, there are a set of “rules” that the tool consults while parsing a user’s spreadsheet. The tool checks for best practices and reports back; this is not in any way set by the DataUp team. 

*There is sparse information on uptake or the impact of uptake. The number of downloads are listed but not how many datasets were uploaded to the repository using DataUp. Despite the excellent requirements survey and user engagement, there is no evaluation of DataUp’s use. What is the difference in uptake between the Excel and web-based version?*

Unfortunately we don’t have access to this information, nor do we believe it is being collected by the service. We are limited in our ability to modify the code base to obtain these metrics since our technical team is not familiar with the Microsoft Azure service or the C#/.NET code base. 

We can cite the number of downloads of the add-in and the number of datasets uploaded to ONEShare, however this does not give us metrics that allow comparison of add-in versus web application. 

More broadly, we are not able to extensively evaluate the use of DataUp because of our limited ability to access user information. Based on the number of submissions to ONEShare, uptake of the tool by researchers has been minimal but steady. 

*There is no related work section. The only related work is RightField (reference17) but what RightField does and how it relates to DataUp is not mentioned. Similar tools to DataUp such as ISAtool Suite and Ontomaton are not mentioned.*

We have added a paragraph to the introduction on existing work in this area.

*The survey appears USA-centric. EZID is only available in the USA. DataUp currently works with DataONEShare. Can and how DataUp be adapted for use in other repositories? Can it reuse infrastructure that is not US based?*

The code is openly available for anyone to use, and the CDL encourages other organizations to take the code and deploy their own instances of DataUp. The identifier provided for a dataset is via the repository; ONEShare is a US repository with connections to the EZID identifier service via the CDL. If other repositories deployed instances of DataUp, the identifier schema would be specific to their existing system.
